# Magnetic Resonance Cholangiopancreatography in the Diagnosis of Haemobilia

**DOI:** 10.1155/2013/792109

**Published:** 2013-12-22

**Authors:** Ines Casazza, Mara Angela Guglietta, Giuseppe Argento

**Affiliations:** Deptartment of Radiology, Sant'Andrea Hospital, Sapienza University, Rome, Italy

## Abstract

Haemobilia is a rare cause of unrecognized gastrointestinal bleeding and is hard to diagnose. Through the present case report we aim to corroborate magnetic resonance relevance in the evaluation of biliary system and bile features, investigating on its role in patients with acute biliary diseases. We report a case of a Caucasian 48-year-old man who was admitted due to abdominal pain and fever. After an ultrasonography exam we detected multiple cysts in the hepatic left lobe: imaging features, laboratory findings, and patient past work experience (woodcutter) suggested a diagnosis of hepatic Echinococcosis. Once surgery decision was taken, patient underwent an intervention of cystopericystectomy. On the 8th postoperative day, the procedure was complicated by black stool, jaundice, and severe anaemia. Acomputed tomography revealed an inhomogeneous collection with some air bubbles in the area of previous surgical intervention, but it was not able to solve the diagnosis question. At this stage a magnetic resonance study was mandatory. On T2-weighted images we observed an expanse gallbladder with hypointense intraluminal material and a considerable intrahepatic biliary system dilatation due to bloody material. On the basis of these examination results, we supposed haemobilia arising from previous surgical intervention. A therapeutic endoscopic retrograde cholangiopancreatography procedure led to decompression of biliary system through a major papilla sphincterotomy with spillage of bile mixed with blood clots.

## 1. Introduction 

Magnetic resonance cholangiopancreatography (MRCP) has an important role in identifying acute and chronic disorders of gallbladder and intra- and extrahepatic biliary system. In most cases, computed tomography signs are not conclusive in the assessment of alterations in abnormal bile. Moreover, MRCP advantages over other imaging modalities include a comprehensive assessment of parenchymal structures, ductal structures, vessels, extrahepatic tissues, and fluid collections. Finally, MRCP is noninvasive, does not result in any radiation exposure, and is a multiplanar exam. Thus, for the latter, image can be projected in several planes and coronal images can depict the entire biliary/pancreatic system.

## 2. Case Report

**Figure 1:**
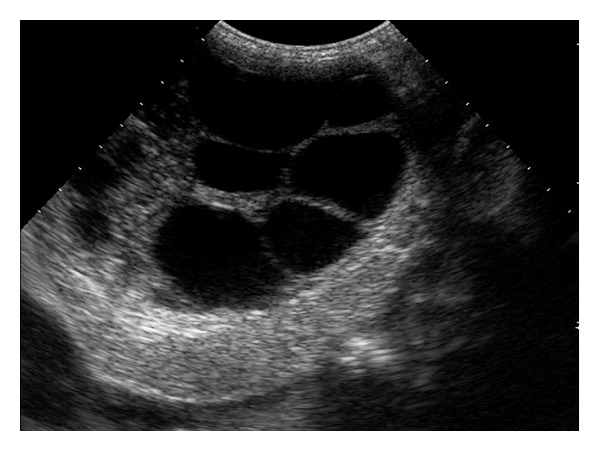
Ultrasonography scan of liver showing multilocular anechoic cystic lesion made up of multiple cysts that ranged from 10 to 45 mm in diameter occupying whole left hepatic lobe. Biliary and vascular structures are displaced but not involved.

**Figure 2:**
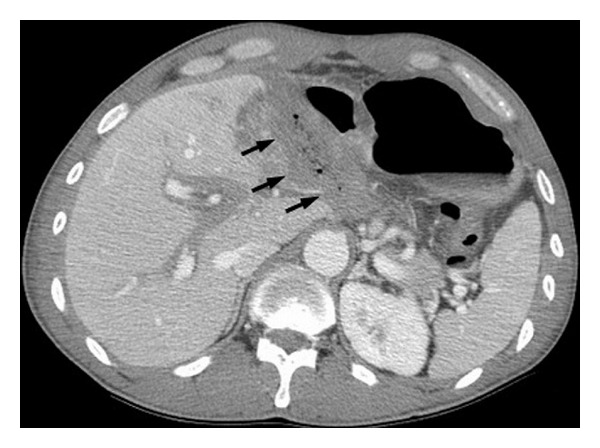
Abdominal computed tomography scan obtained after administration of contrast material shows mild biliary tree dilatation and a fluid collection with some air bubbles within.

**Figure 3:**
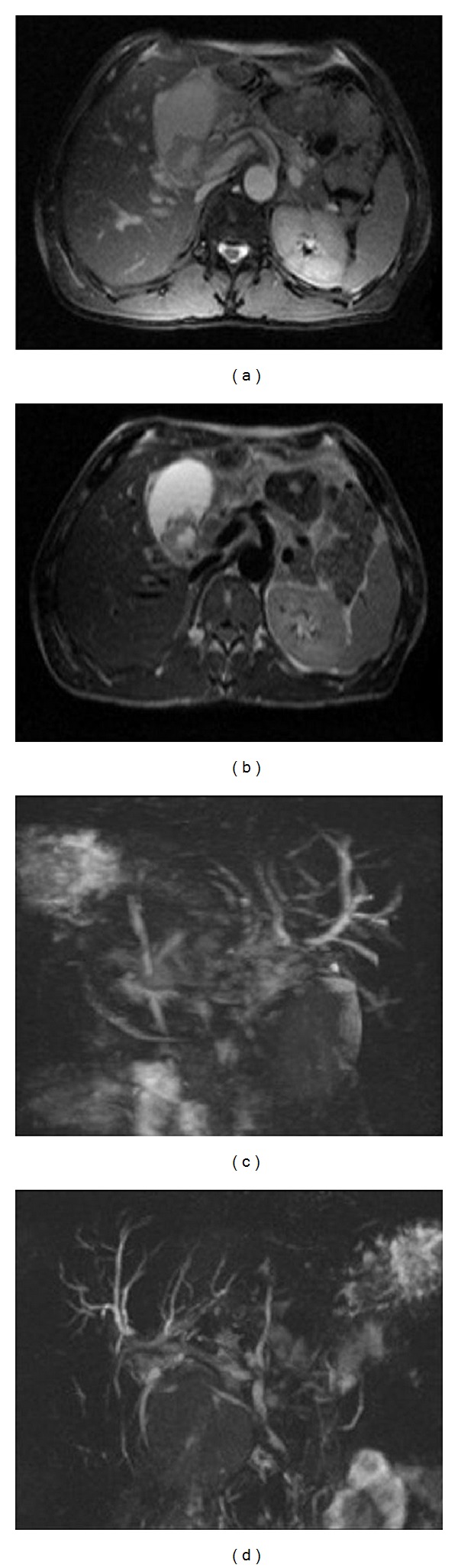
(a), (b) Axial fat saturated T2-weighted magnetic resonance images showing bile ducts dilatation and a fluid collection around left hepatic lobe and around gallbladder; inside the main bile duct and in the gallbladder note materials with hypointense signal due to blood clots. (c), (d) 2D and 3D haste MIP images showing low signal intensity of blood clots inside dilatated bile tree, sign of haemobilia, and inflammatory narrowing of distal choledocus.

**Figure 4:**
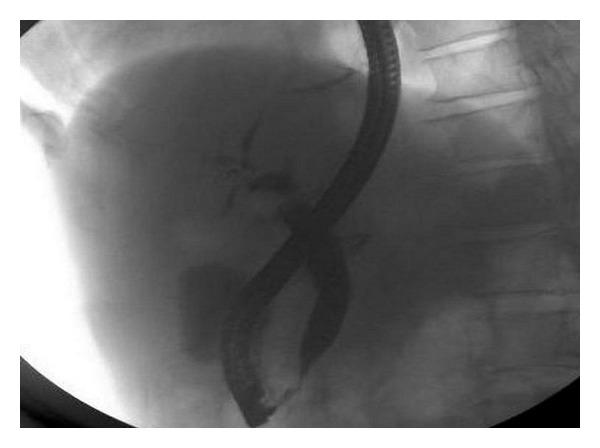
Endoscopic retrograde cholangiopancreatography. After endoscopic maior papillary sphincterotomy and drainage of blood clots mixed up with bile, cholangiography with iodinated contrast media retrograde injection shows mild bile ducts dilatation.

A 48-year-old white man with abdominal pain and fever (above 39°C) came to our Emergency Ward. The patient was in a status of good health since three weeks before admission, when he was affected by postprandial vomiting and epigastric pain. On examination he showed a splitting of the first heart sound and a liver two fingers beneath the right costal margin; the rest of examination was normal. Laboratory tests revealed normal value of blood count, except eosinophilia (1305 cells/mm^3^ that is about 11,3% of the white blood cells); hepatic parameters, glycemia, and renal function were also in range. Patient's past medical history was unremarkable: he had only undergone an appendicectomy several years before. Patient underwent ultrasonography of the abdomen that revealed a septated cystic lesion with “honeycomb-like” structures localized in the hepatic left lobe (Figure 1). Wide parenchymal lesion caused constriction and dislocation of suprahepatic veins and left branch of portal vein. Given the mass features a provisional diagnosis of hydatid cyst was made, supported also by presence of echinococcal antibodies detected by indirect hemagglutination and by patient history (he referred that he was a woodcutter). The patient underwent a surgical approach with a radical procedure of cysto-pericystectomy. Histopathological examination confirmed the diagnosis of Echinococcosis. On the 8th postoperative day, patient had referred intensive epigastric pain radiating to right shoulder and jaundice (conjugated bilirubin 4.2 mg/dL) with severe anaemia (that necessitated blood transfusions) and black stool. A computed tomography study of the abdomen revealed a mild bile duct dilatation and an inhomogeneous collection with air bubbles in the area where previous surgical treatment was made (Figure 2). Since computed tomography findings were inconclusive, MRCP was performed. On magnetic resonance images there was material into bile ducts with mixed signal intensity on T2-weighted images according to the presence of blood products in various stages of breakdown (Figure 3). On MRCP 3D images some filling defects could be detected. All these features were compatible with the diagnosis of haemobilia. Our patient underwent a therapeutic endoscopic retrograde cholangiopancreatography procedure that confirmed hepatobiliary tract dilatation because of obstruction by blood clots (Figure 4).

## 3. Discussion 

Haemobilia is defined as bleeding into the bile ducts and/or gallbladder [1]: it is the result of an abnormal communication between splanchnic circulation and bile ducts. The anatomic origin of haemobilia can be identified in liver parenchyma, extrahepatic bile ducts, gallbladder, or pancreas [2, 3]. The first case of haemobilia was described in 1654 by Francis Glisson, the Regius Professor of Physic in Cambridge [4], while the term “haemobilia” was first coined by Sandblom in 1948 [5]. The main cause of haemobilia is iatrogenic trauma, followed by gallstone disease, accidental trauma, acalculous inflammatory diseases, malignant tumors (such as hepatocellular carcinoma and cholangiocarcinoma), vascular anomalies (aneurysms and arteriovenous malformations), liver abscess, radiofrequency ablation, parasitic infection, and systemic autoimmune diseases [3, 4, 6–8]. Its incidence increased due to spread of the interventional liver procedures. Classical onset with a characteristic triad of jaundice (due to bile duct obstruction by clots), melena, and right-sided epigastric pain is not frequent. Most often haemobilia can have only one of these signs or other signs such as biliary colic, fever, and anaemia [3, 4]. It is a rare cause of upper gastrointestinal hemorrhage. Haemobilia can rarely lead to pancreatitis, cholecystitis, and cholangitis [9–11]. We define mild to moderate haemobilia as a bleeding lasting shorter than 48 hours, without the need for blood transfusions, while hemodynamic instability or haemorrhage necessitating transfusion define severe haemobilia [7]. The gold standard for studying haemobilia is angiography, but it can be negative if there is not an active bleeding, although the advantage of this procedure is the possibility of an embolization that is one of the treatments in the course of haemobilia [12]. The most appropriate investigation is choosen according to the patient, suspected aetiology, and the seriousness of illness. In case of upper gastrointestinal bleeding oesophagogastroduodenoscopy is the most appropriate investigation. If haemobilia has a traumatic origin, then it is useful to perform an oesophagogastroduodenoscopy than a computed tomography, and if the diagnosis is not achieved, it is necessary to have an angiography. Ultrasonography is a nonspecific exam it is not useful soon after a bile duct haemorrhage because hepatobiliary tract echogenicity is similar to that of liver parenchyma, whereas after 24 h–48 h bile ducts become hyperechogenic with respect to the entire liver [13, 14]. Computed tomography has a high level of specificity but a low sensibility when dealing with the evaluation of haemobilia. In particular, both in computed tomography and angiography, intermittent bleeding and low hemorrhagic flow can produce false negatives. In our case study, on the one hand, computed tomography registered the presence of an inhomogeneous collection with air bubbles in the area where previous surgical treatment was made, but no bleeding source was identified. On the other, magnetic resonance detected more specific features. In particular, T2-weighted images revealed presence of hypointense material in the gallbladder, in intrahepatic bile ducts, and in common hepatic duct. Bile that is just made has features as free water that is to say it is hypointense on T1-weighted images and bright on T2-weighted images [15]. In the fasting state both the T1 and T2 relaxation times are shortened [16]. Hemorrhagic bile has a signal of low intensity in the lower-dependent layer on both axial heavily T2-weighted images and fat-suppressed T2-weighted images. Considering magnetic resonance images, haemobilia is identifiable in a signal of mixed intensity on T2-weighted images and clots appear as filling defects in the gallbladder and in bile ducts. In the light of these evidence, our magnetic resonance study was consistent with haemobilia.

## 4. Conclusion 

This case illustrates the different roles of imaging studies in differential diagnosis of bile disorders and in particular in the assessment of haemobilia. Magnetic resonance provides better tissue characterization than computed tomography or ultrasonography, so its use should be considered in the evaluation of hemodynamically stable patients with suspected nontraumatic haemobilia.
